# Ligand-receptor interaction atlas within and between tumor cells and T cells in lung adenocarcinoma

**DOI:** 10.7150/ijbs.42080

**Published:** 2020-05-18

**Authors:** Zhencong Chen, Xiaodong Yang, Guoshu Bi, Jiaqi Liang, Zhengyang Hu, Mengnan Zhao, Ming Li, Tao Lu, Yuansheng Zheng, Qihai Sui, Yong Yang, Cheng Zhan, Wei Jiang, Qun Wang, Lijie Tan

**Affiliations:** 1 Department of Thoracic Surgery, Zhongshan Hospital, Fudan University, No. 180, Fenglin Road, Shanghai, 200032, China; 2 Shanghai Medical College, Fudan University, Shanghai 200032, China; 3 Department of Thoracic Surgery, The Affiliated Suzhou Hospital of Nanjing Medical University, Suzhou 215001, China

**Keywords:** Lung adenocarcinoma, Single-cell RNA-seq, Cell-to-cell interactions, Machine learning, Survival

## Abstract

**Purpose**: Lung adenocarcinoma (LUAD) is the leading cause of cancer-related deaths worldwide. Although tumor cell-T cell interactions are known to play a fundamental role in promoting tumor progression, these interactions have not been explored in LUAD.

**Methods**: The 10x genomics single-cell RNA sequencing (scRNA-seq) and gene expression data of LUAD patients were obtained from ArrayExpress, TCGA, and GEO databases. scRNA-seq data were analyzed and infiltrating tumor cells, epithelial cells, and T cells were identified in the tumor microenvironment. Differentially expressed ligand-receptor pairs were identified in tumor cells/normal epithelial cells and tumor T cells/non-tumor T cells based on corresponding scRNA-seq and gene expression data, respectively. These important interactions inside/across cancer cells and T cells in LUAD were systematically analyzed. Furthermore, a valid prognostic machine-learning model based on ligand-receptor interactions was built to predict the prognosis of LUAD patients. Flow cytometry and qRT-PCR were performed to validate the significantly differently expressed ligand-receptor pairs.

**Results**: Overall, 39,692 cells in scRNA-seq data were included in our study after quality filtering. A total of 65 ligand-receptor pairs (17 upregulated and 48 downregulated), including LAMB1-ITGB1, CD70-CD27, and HLA-B-LILRB2, and 96 ligand-receptor pairs (41 upregulated and 55 downregulated), including CCL5-CCR5, SELPLG-ITGB2, and CXCL13-CXCR5, were identified in LUAD cancer cells and T cells, respectively. To explore the crosstalk between cancer cells and T cells, 114 ligand-receptor pairs, including 11 ligand-receptor pair genes that could significantly affect survival outcomes, were identified in our research. A machine-learning model was established to accurately predict the prognosis of LUAD patients and ITGB4, CXCR5, and MET were found to play an important role in prognosis in our model. Flow cytometry and qRT-PCR analyses indicated the reliability of our study.

**Conclusion**: Our study revealed functionally significant interactions within and between cancer cells and T cells. We believe these observations will improve our understanding of potential mechanisms of tumor microenvironment contributions to cancer progression and help identify potential targets for immunotherapy in the future.

## Introduction

Lung cancer is the leading cause of cancer-related deaths worldwide and is responsible for more than 1,700,000 new cases every year 1, 2. Lung adenocarcinoma (LUAD), which accounts for more than 50% of all lung cancers, is one of the most important subtypes of lung cancer 1, 3. As an important ingredient of tumor tissues, the tumor microenvironment (TME) plays a fundamental role in promoting tumor progression, including proliferation, invasion, metastasis, and drug resistance 4, 5. Several studies have suggested that T cells, which are closely related to immune therapy and patient survival, represent the most prevalent cell type in the TME of LUAD 6, 7. However, how T cells interact with tumor cells has not been extensively explored.

In recent decades, studies on the expression profile of LUAD have mainly been based on RNA sequencing (RNA-seq) technologies, which detect the gene expression of the sample as a whole. However, in addition to tumor cells, tumor tissues also contain a large number of other cell types, such as macrophage cells, epithelial cells, and T cells, and the gene expression profiles of these cell types vary substantially. Therefore, the percentages of different cell types influence the results of RNA-seq, and it is difficult to investigate interactions among cell subpopulations using RNA-Seq data. Therefore, 10x genomics single-cell sequencing (scRNA-seq), which is focused on the main characteristics of each cell subpopulation and their interaction in the TME, has broad prospects, important applications, and research value 8, 9.

In the present study, scRNA-seq data of LUAD was used to explore significant interactions within cancer cells and T cells in LUAD. Communication between LUAD tumor cells and T cells was also explored. A machine learning model based on ligand-receptor interactions between T cells and LUAD tumor cells was built to predict the survival of patients with LUAD. We believe our results will improve our understanding of communication within and between T cells and LUAD tumor cells of LUAD and its connection with patient survival.

## Results

### LUAD tumor cell and T cell clusters are present in LUAD

**Figure 1:**
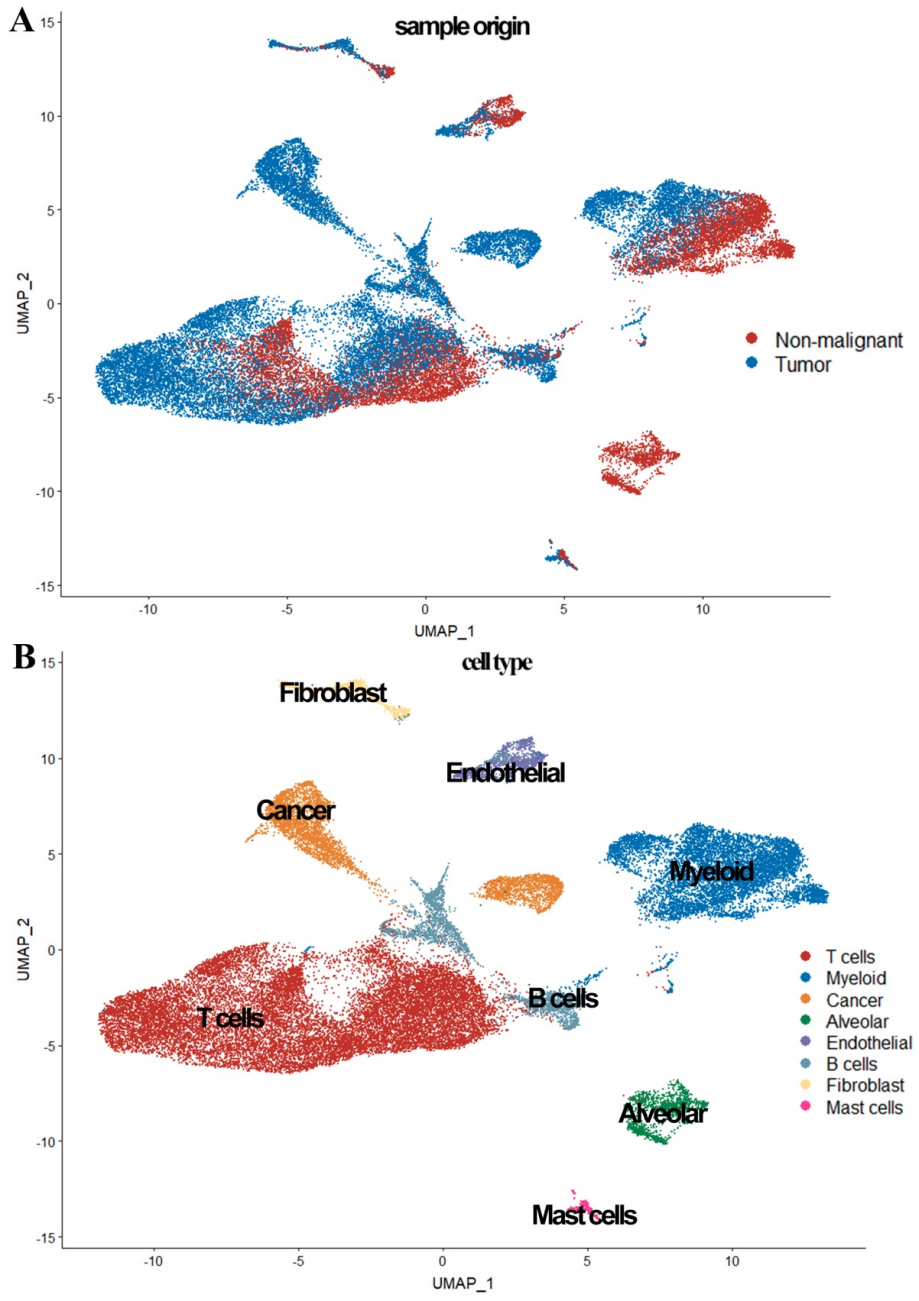
Overview of the 36,095 single cells from six tumor samples and four normal samples. (A) The sample origin of the cells; (B) The cell types identified by marker genes

In the scRNA-seq data analysis, 39,692 cells from five patients (seven tumor samples and four normal samples) were included after quality filtering (Supplementary Figure 1, Supplementary Table 1). Of these, 26,277 cells (66.2%) originated from LUAD and 13,375 (33.8%) originated from normal lung tissues (Figure 1). As shown in Figure 1, 39,692 cells were classified into nine clusters by PCA and UMAP clustering methods; subsequently, these identified cell clusters were assigned to known cell types via marker genes.

**Figure 2:**
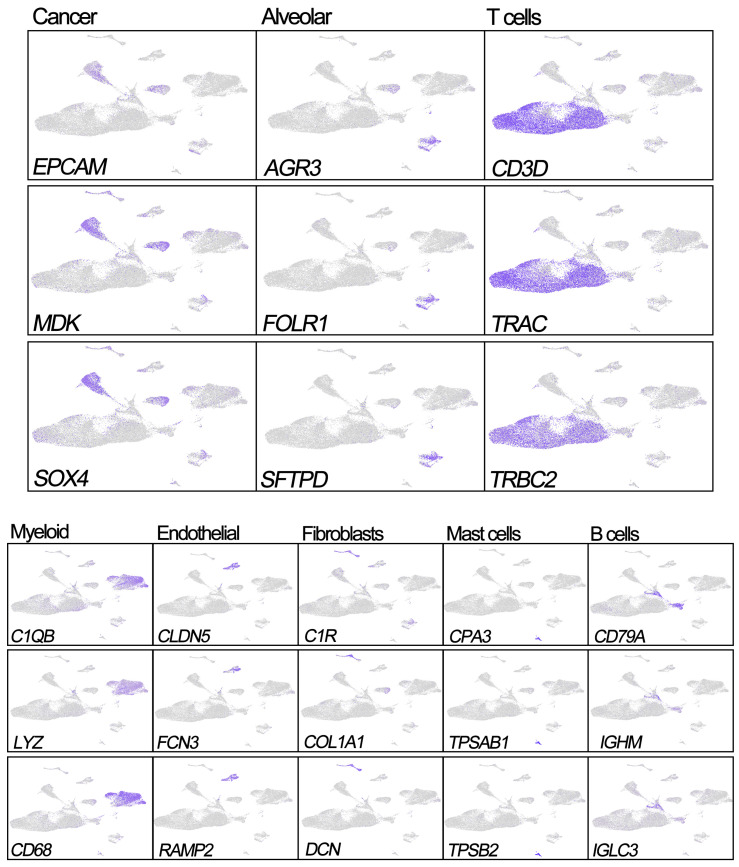
Expression of marker genes for the cell types.

Previous studies have reported that EPCAM, MDK, and SOX4 are tumor cell markers, while FOLR1, SFTPD, and AGR3 are epithelial cell markers 6, 10, 11. To identify the tumor cells and non-tumor lung cells, we first mapped the expression of six genes (FOLR1, AGR3, and SFTPD for normal hung lung cells, and EPCAM, MDK, and SOX4 for cancer cells) to each cluster to identify the cell types in our study. We noticed the 'Alveolar cluster' is mainly made up of cells from normal tissue, while the 'Cancer cluster' is mainly consisting of cells from tumor samples (Figure 1). We also found CD3D, TRAC, and TRBC2, which are considered to be T cell markers, were highly expressed in the 'T cells cluster' 6, 10, 11. Therefore, we confirmed that the 'Alveolar cluster' was normal epithelial cells (1,483 cells, 11.1% in normal samples) and the 'Cancer cluster' was LUAD tumor cells (4,637 cells, 17.6% in tumor samples). And we identified 18,824 T cells (6,233 [46.6%] in non-neoplastic and 12,527 cells [47.7.4%] in neoplastic samples) in our study. Detailed information of the marker genes for each cluster is shown in Figure 2.

**Figure 3:**
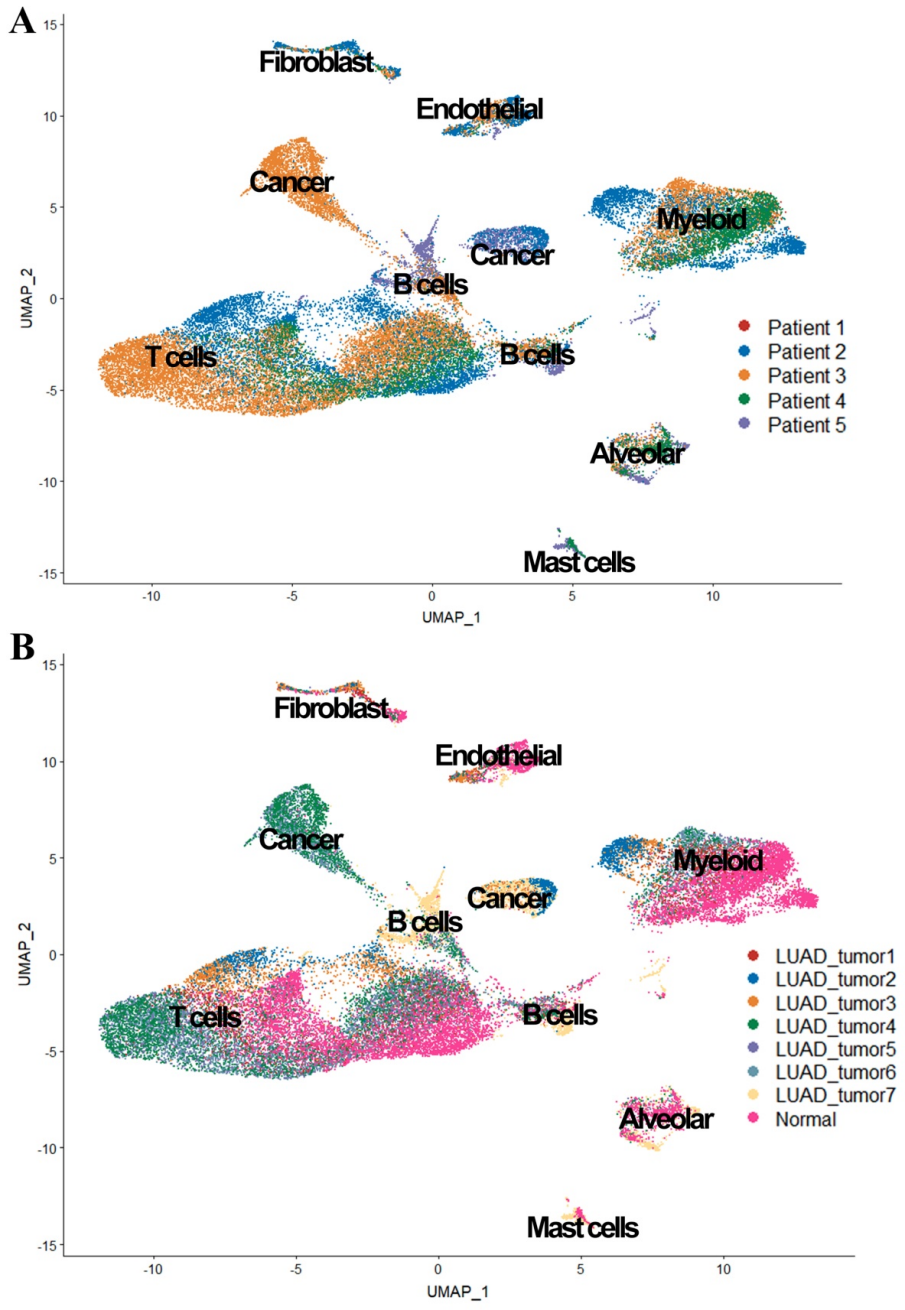
UMAP plot of the 36,095 single cells. (A) Cells grouped by the origin of patients; (B) Cells grouped by the origin of samples.

We also observed in Figure 3, tumor cells were mainly grouped by its source, while cells from patient 1 and patient 4 were scattered in different cell types (expect 'Cancer cluster'), suggesting the intertumor heterogeneity in our study.

### Expression correlation analysis reveals significant ligand-receptor pairs in LUAD tumor cells

**Figure 4:**
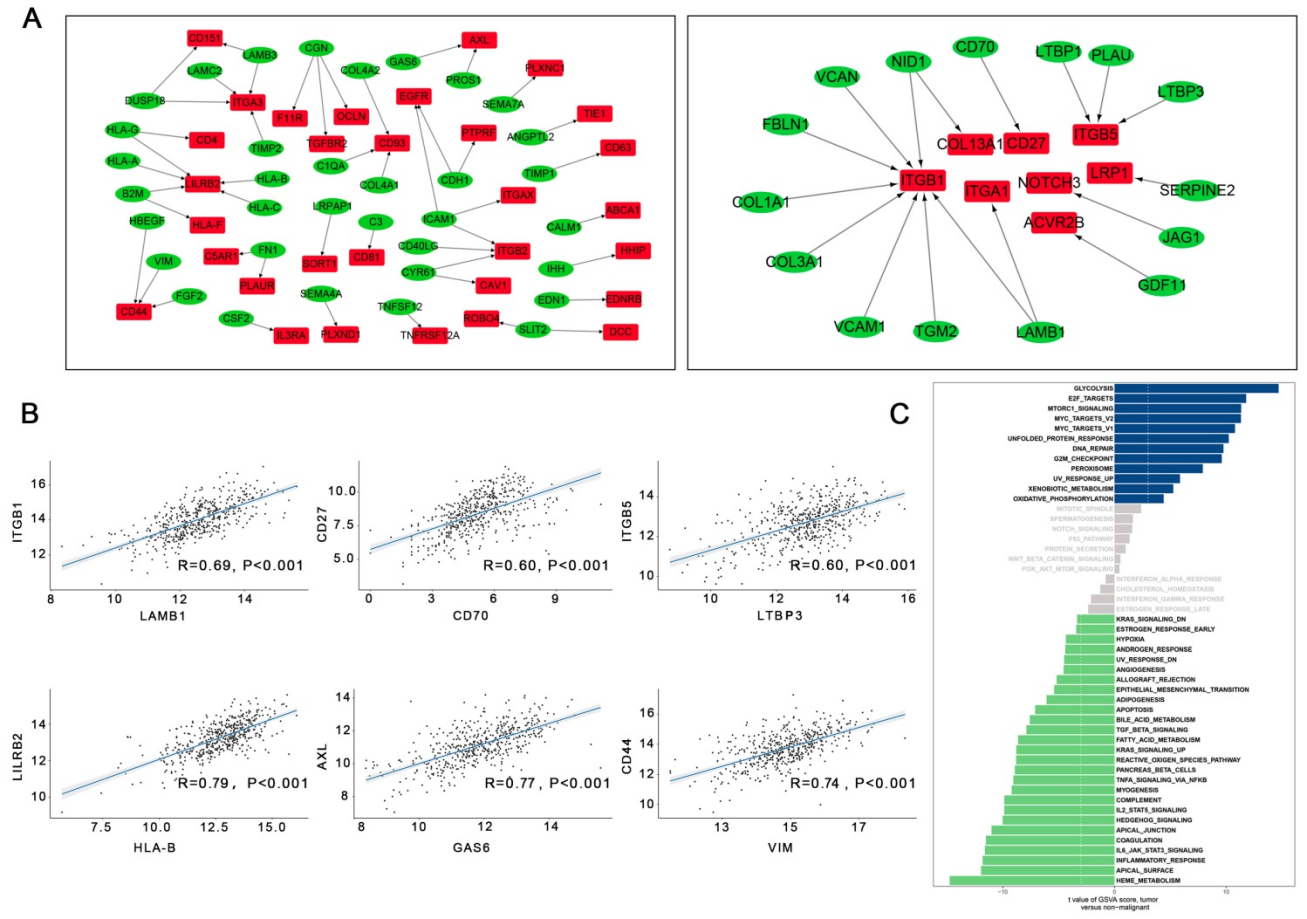
The intracellular ligand-receptor signaling network identified in LUAD tumor cells. (A) Ligand-receptor pairs of intracellular signals inside LUAD neoplastic cells. Green dots stand for ligands and red dots stand for receptors; (B) Spearman's correlation coefficients of ligand-receptor pairs in TCGA LUAD dataset; (C) GSVA analysis of the hallmark pathways in LUAD tumor cells (tumor versus non-malignant).

A total of 8849 differentially expressed genes were detected between LUAD tumor cells and epithelial cells (Supplementary Table 2). Overall, 56 upregulated and 167 downregulated pairs in which the receptors and ligands were increased or decreased simultaneously were identified in LUAD tumor cells. TCGA dataset was used to calculate Spearman's correlation coefficients to explore the co-expression of a ligand and its corresponding receptor. As shown in Figure 4A, with strict criteria of coefficient > 0.4 and P < 0.05 in Spearman's correlation rank test, 17 upregulated and 48 downregulated pairs were identified (Supplementary Table 2, Figure 4A).

In our study, the most relevant upregulated pair was LAMB1 and its receptors ITGB1, followed CD70-CD27, and LTBP3-ITGB5 (Figure 4B). Interestingly, upregulation of LAMB1 and its receptors ITGB1 has been reported to promote the development of tumors by destroying endothelial cell function 12; a previous study suggested that upregulation of LAMB1-ITGB1 plays an important role in the proliferation, adhesion, migration of human^13^. Many studies have also shown that upregulation of CD70-CD27 is related to T and B cell activation 14. Moreover, the top three downregulated pairs in LUAD tumor cells were HLA-B and its ligand LILRB2, GAS6-AXL, and VIM-CD44 (Figure 4B). Seike et al. reported that AXL and GAS6 co-expression in LUAD was strongly related to survival and could serve as a prognostic classifier 15.

A direct comparison of LUAD tumor cells and normal epithelial cells was performed and is shown in Figure 4C. Glycolysis, e2f targets, mtorc1 signaling, Myc targets v2, and Myc targets v1 were the top five enriched pathways in LUAD tumor cells. Other enriched pathways such as e2f were also associated with each step of tumorigenesis. Overexpression of e2f in the lung can lead to an accelerated cell cycle and increased cell proliferation, suggesting that downregulation of e2f might be a potential therapeutic target in LUAD 16.

### Intracellular network of tumor T cells

Previous studies had reported that tumor tissue contains a large number of immune cells and T cells represented a large proportion in tumor tissue 17.To better show the proportion of T cells in the TCGA database, CIBERSORT also had performed in our study and our results revealed that T cells took a large proportion of all cells in LUAD (Supplementary Figure 2) 18. Therefore, the TCGA database was used to analyze the ligand-receptor interactions among T cells in our study.

**Figure 5:**
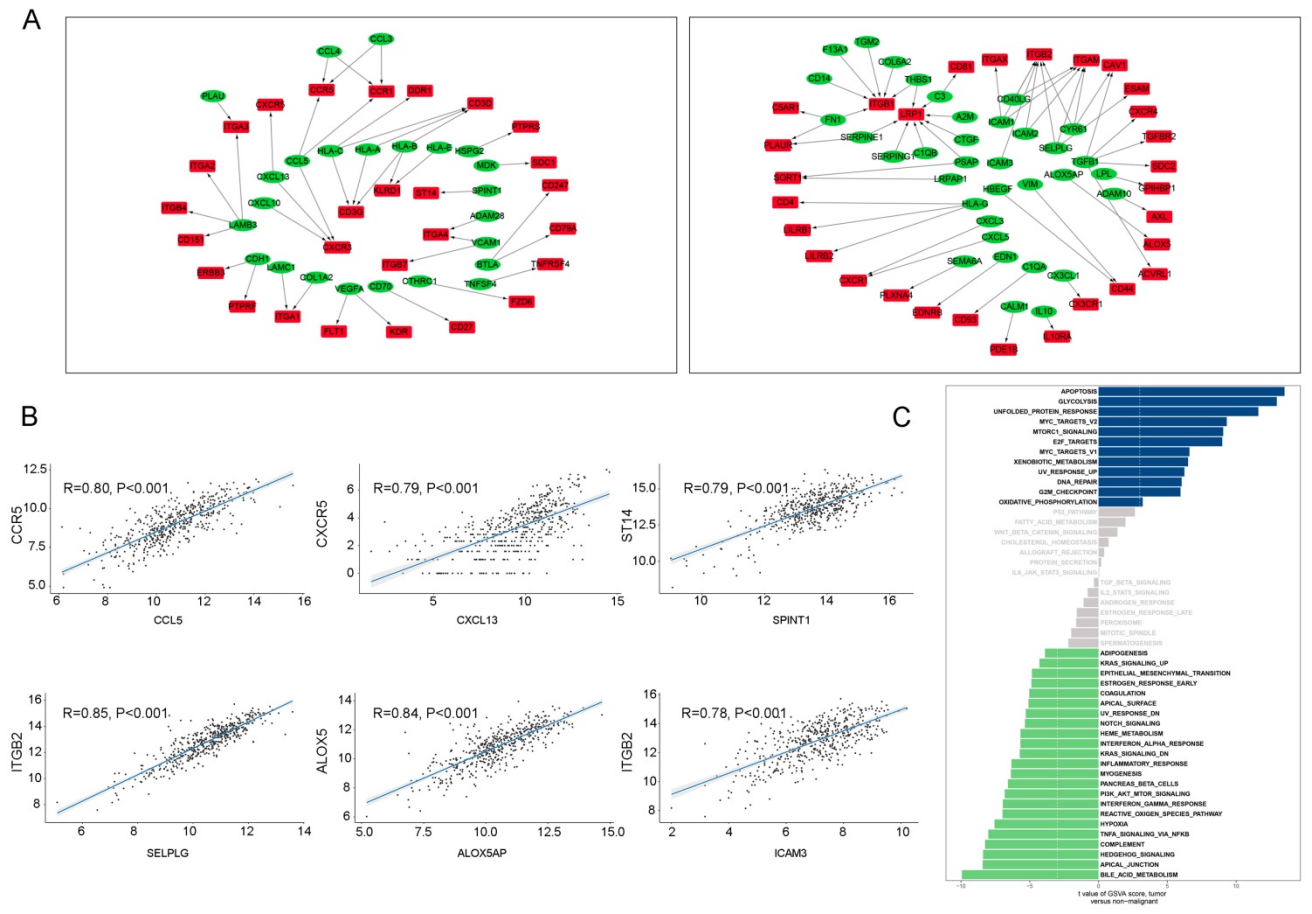
The intracellular ligand-receptor signaling network identified in T cells. (A) Ligand-receptor pairs of intracellular signals inside T cells. Green dots stand for ligands and red dots stand for receptors;(B) Spearman's correlation coefficients of ligand-receptor pairs in TCGA LUAD dataset.; (C) GSVA analysis of the hallmark pathways in T cells (tumor versus non-malignant).

To investigate the intracellular network of tumor T cells, 5255 differentially expressed genes between tumor T cells and non-tumor T cells were selected in our study for downstream analysis (Supplementary Table 2). The numbers of upregulated and downregulated pairs in tumor T cells were 76 and 122, respectively, in our initial selection. After Spearman's correlation rank test, 41 upregulated and 55 downregulated pairs were detected (Figure 5A, Supplementary Table 2). Additionally, upregulated and downregulated pairs ranked in the top three were CCL5-CCR5, CXCL13-CXCR5, and SPINT1-ST14; and SELPLG-ITGB2, ALOX5AP-ALOX5, and ICAM3-ITGB2, respectively (Figure 5B).

CCL5 has been hypothesized to be highly expressed in lung cancer and co-expression of CCL5-CCR5 could promote tumor invasion and metastasis by activating PI3K/Akt signaling 19, 20. When compared the pathway expression levels in tumor-derived T cells and non-tumor T cells (Figure 5C), we noticed that tumor-associated increases in disorder of the cell cycle (apoptosis, unfolded protein response, DNA repair, and G2M checkpoint), cell proliferation (myc targets, mtorc1 signaling, and e2f targets), and biomass production (glycolysis and xenobiotic metabolism).

Additionally, many previous studies had reported CCL5 and CXCL13 were mainly expressed in the tumor cells 19, 21. In our study, our results also revealed that compared with normal lung epithelial cells, CCL5 and CXCL13 had higher expressions in tumor cells. To our surprise, we also found CCL5 and CXCL13 had the highest expression levels in tumor-associated T cells, which indicates that CCL5 and CXCL13 may play important roles in tumor immunity (Supplementary Figure 3). Further research needs to perform to investigate the expressions of CCL5 and CXCL13 in tumor and T cells.

### Crosstalk between LUAD tumor cells and T cells

**Figure 6:**
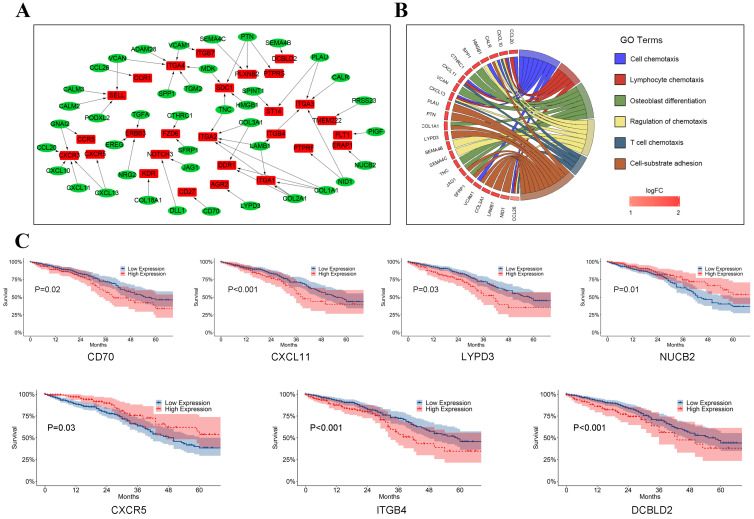
The crosstalk from LUAD tumor cells to T cells. (A) Ligand-receptor pairs of the signaling network from LUAD tumor cells to T cells. Green dots stand for ligands highly expressed in LUAD tumor cells and red dots stand for receptors highly expressed in T cells; (B) Significantly gene functional enrichment analysis for ligand-receptor pairs in the crosstalk from LUAD tumor cells to T cells; (C)Kaplan-Meier survival analysis for ligand-receptor pairs in TCGA LUAD dataset.

To assess crosstalk between LUAD tumor cells and T cells, 59 ligand-receptor pairs in which ligands and receptors were separately highly expressed in LUAD tumor cells and T cells were selected in our study (Figure 6A, Supplementary Table 2). Interestingly, ligands encoding collagens I and III (e.g., COL1A1-DDR1, COL2A1-ITGA1, and COL3A1-ITGA2), which act as ligands, were found highly expressed in LUAD tumor cells. Wyckoff et al. reported that cancer cells might migrate along the collagen fiber, suggesting that these ligand-receptor pairs may promote the migration of tumor cells in LUAD 22. CXCR3 and CXCR5 (e.g., CXCL10-CXCR3, CXCL11-CXCR3, and CXCL13-CXCR5), which belong to the CXC chemokine receptor family, were found highly expressed in tumor T cells. CXCR3 plays an important role in promoting tumor growth and metastasis by interfering with T cell function and an antagonist of CXCR3 may inhibit tumor metastasis 23, 24. Gene functional enrichment analysis in the present study suggested that genes involved in crosstalk between LUAD tumor cells and T cells were likely mainly related to the chemokine response, which has been linked to metastasis, tumor angiogenesis, and immune escape (Figure 6B) 25.

In survival analyses of ligands highly expressed in LUAD tumor cells, we observed that patients with high expression of CD70 (P = 0.02), CXCL11 (P < 0.001), and LYPD3 (P = 0.03) were related to poorer prognosis while patients with high expression of NUCB2 (P = 0.01) had significantly better prognosis (Figure 6C). As shown in Figure 6C, CXCR5 (P = 0.03), ITGB4 (P < 0.001), and DCBLD2 (P < 0.001), which act as receptors in T cells, were significant prognostic indicators for TCGA LUAD dataset.

### Cell-cell communication between T cells and LUAD tumor cells

**Figure 7:**
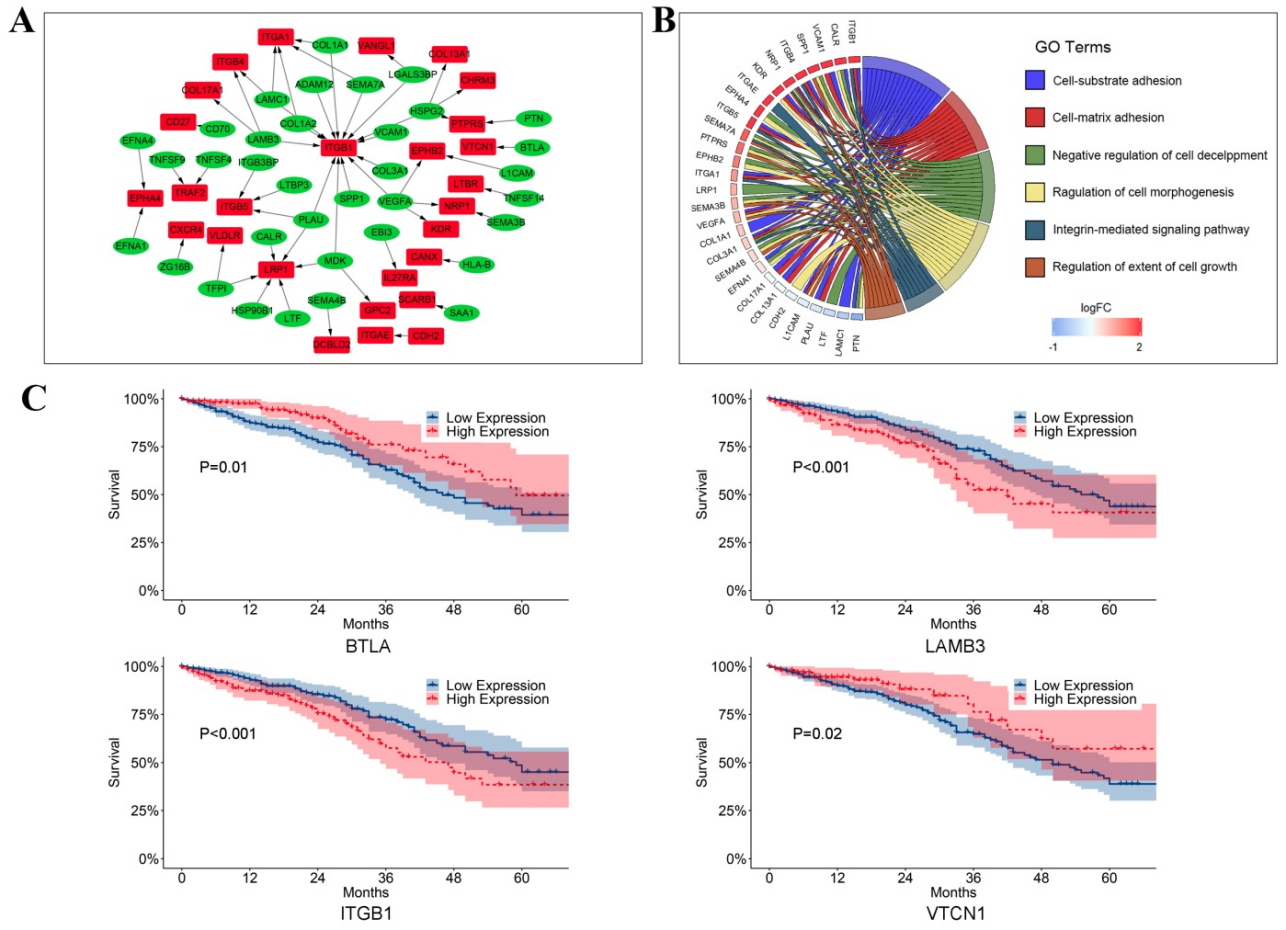
The crosstalk from T cells to LUAD tumor cells. (A) Ligand-receptor pairs of the signaling network from T cells to LUAD tumor cells. Green dots stand for ligands highly expressed in T cells and red dots stand for receptors highly expressed in LUAD tumor cells; (B) Significantly gene functional enrichment analysis for ligand-receptor pairs in the crosstalk from T cells to LUAD tumor cells; (C) Kaplan-Meier survival analysis for ligand-receptor pairs in TCGA LUAD dataset.

To analyze how T cells communicated with LUAD tumor cells via ligand-receptor pairs, Spearman's analyses were conducted in our study. Then 55 ligand-receptor pairs were selected in our study (Figure 7A, Supplementary Table 2). Interestingly, the tumor necrosis factor superfamily (e.g., TNFSF4-LTBR, TNFSF9-TRAF2, and TNFSF14-TRAF2) was highly expressed in T cells. A previous study reported that TNFSF4 was predominantly expressed on activated CD4+ T cells and could activate CD4+ T cells by secreting signals after binding to its receptor. Furthermore, we identified that members of the integrin receptor family (e.g., LAMB3-ITGB1, LTBP3-ITGB5, and COL1A1-ITGA1) were highly expressed in LUAD tumor cells. Dingemans et al. reported that the integrin family might play a role in lymph node metastasis of LUAD by activating endothelial cells during tumor angiogenesis 26. Additionally, these ligand-receptor pairs are primarily associated with invasion and proliferation (Figure 7B). In our study, to our surprise, we found that some ligands and receptors were highly expressed in both tumor cells and T cells, such as collagen and integrin family genes, such as COL1A1 and ITGB1 (Supplementary Figure 4). It suggests that in the process of tumor development, many ligand-receptor pairs were simultaneously activated in many cell types, which may regulate by the upstream regulators, further studies need to conduct to explore the cor- ligand-receptor pairs.

To better compared the ligand-receptor pairs in LUAD tumor cells and T cells, we compared the expression of ITGB1, LAMB1, CD70, CD27, CXCR5, CXCL13, ITGB4, and CCL5 in different patients. As shown in Supplementary Figure 5, compared with normal samples, the expression of these eight genes was increased in tumor samples in general. Intriguingly, we also observed that the relative expressions of these genes were different in different samples, even if samples were derived from the same patient. For example, CXCL13 had the highest expression level in Sample 3 and had the lowest expression level in Sample 2. Additionally, to comprehensively analyze these pairs in our study, we also compared the expression of ITGB1, LAMB1, CD70, CD27, CXCR5, CXCL13, ITGB4, and CCL5 in the pairs of tumor and corresponding normal samples (Supplementary Figure 6). Our results revealed that in different samples and different patients, the activated ligand-receptor pairs can be different, suggesting the heterogeneity in the tumor.

To confirm the activation of pairs in the LUAD tumor cells and T cells, the downstream elements (ARRB2 and JAK3 were the downstream elements for the receptors of CXCR3 in T cells, PTK2 and ACTG1 were downstream elements for ITGB4) of these pairs were selected in our study. As shown in Supplementary Figure 7, ARRB2 and JAK3 were highly expressed in T cells, while PTK2 and ACTG1 were mainly enriched in the 'Cancer cluster. These results revealed downstream elements of CXCR3 and ITGB4 are activated, which proved the reliability of our study.

We also observed that CD70, ITGB4, and DCBLD2 were significant prognostic indicators in crosstalk and cell-cell communication between LUAD tumor cells and T cells (Figure 7C). As shown in Figure 7C, we also found that BTLA (P = 0.01), LAMB3 (P < 0.001), ITGB1 (P < 0.001), and VTCN1 (P = 0.02) were significantly related to survival.

### Prognostic model based on machine learning

**Figure 8:**
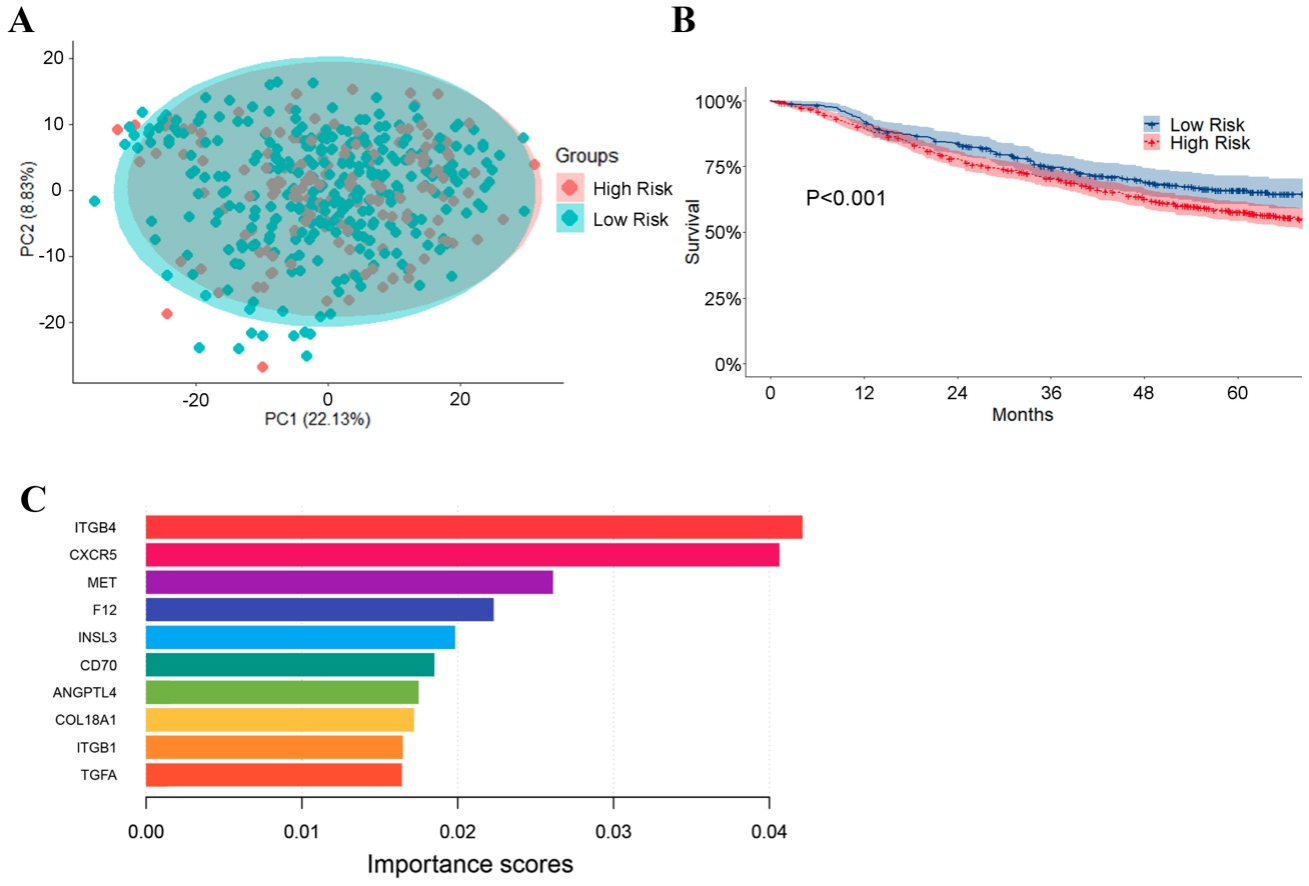
Prognostic predictor for LUAD patients based on XGBoost. (A) PCA of low risk (stage IA) and high risk (stage IB-IV) LUAD groups based on the genes of ligand-receptor pairs; (B) The performance of the prognostic predictor Kaplan-Meier survival analysis for the patients in GEO dataset (n = 1,063 and P-value<0.001); (C) Importance rank of the top 10 genes in the prognostic classifier. Importance scores stand for the importance of genes in the predicting model.

As shown in Figure 8, low risk (stage IA, 127 cases) and high risk (stage IB-IV, 333 cases) groups of TCGA LUAD dataset mixed in PCA (Figure 8A), suggesting that there is little difference in the expression of ligand-receptor pairs between these two groups. A machine-learning model was built based on these ligand-receptor genes. The precision value, recall value, and F1-score of the prognostic model were 0.76, 0.73, and 0.74, respectively.

Subsequently, the GEO dataset (GSE30219, GSE31210, GES3141, GSE37745, GSE50081, and GSE68465), which included 1,063 patients, was used to validate our prognostic model. As shown in Figure 8B, patients in the GEO dataset were divided into high-risk and low-risk groups. Overall survival was significantly different between the high-risk and low-risk groups (P < 0.01). Strikingly, we also revealed ITGB4, CXCR5, and MET as the top three important genes in the prognostic model (Figure 8C).

### Flow cytometry and qRT-PCR

**Figure 9:**
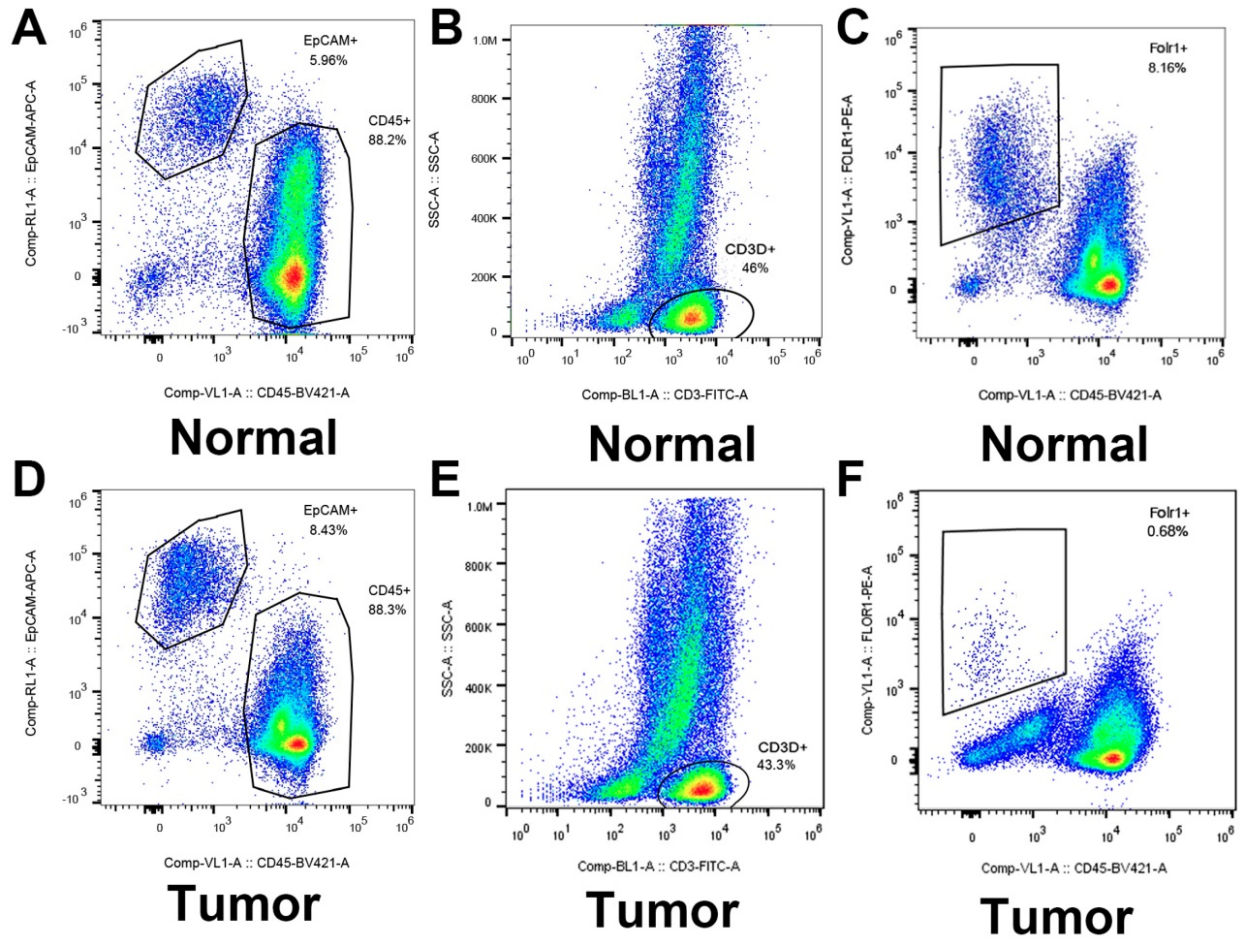
Identified and sorted the key genes expression in epithelial cells (LUAD tumor cells and Alveolar cells) and T cells (Tumor T cells and Non-tumor T cells) by flow cytometry.

**Figure 10:**
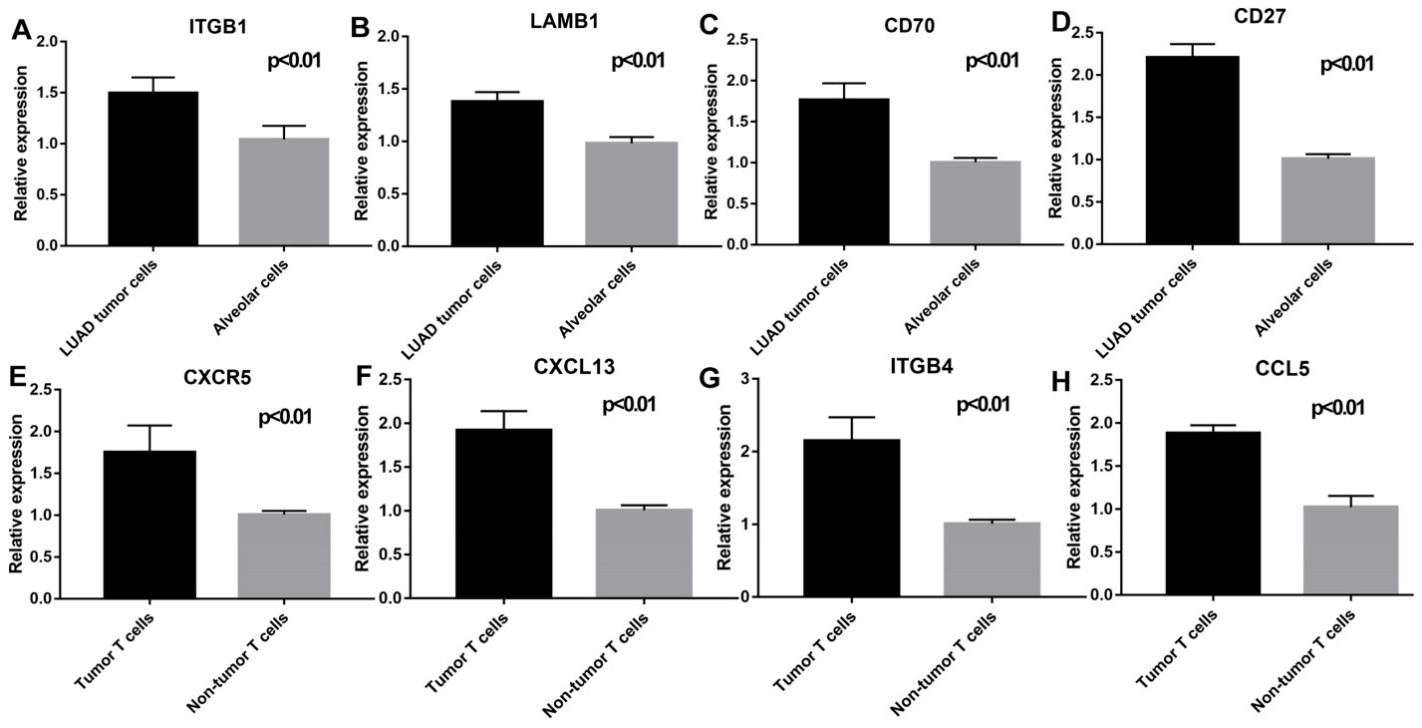
Validation of the key genes expression in epithelial cells (LUAD tumor cells and Alveolar cells) and T cells (Tumor T cells and Non-tumor T cells). ITGB1 (P < 0.01), LAMB1 (P < 0.01), CD70(P < 0.01), and CD27 (P < 0.01) were highly expressed in LUAD tumor cells; CXCR5 (P < 0.01), CXCL13 (P < 0.01), ITGB4 (P < 0.01), and CCL5 (P < 0.01) were highly expressed in tumor T cells.

As shown in Figure 9, flow cytometry was performed to validate epithelial cells marked with EPCAM and FOLR1, T cells marked with CD3D in LUAD and non-malignant lung samples. qRT-PCR was performed to detect differences in gene expression levels between cancer and alveolar cells in malignant and normal lung tissues, as well as the difference between T cells in the tumor and normal samples. Besides, as shown in Supplementary Figure 8, we noticed that compared with tumor lung tissues, EPCAM+/FOLR1+ cells had a much larger proportion in normal samples (48.3% vs.3.91%, 65.4% vs.13.3%, and 68.3% vs.1.08%, in CD45- cells respectively), while there are less EPCAM+/FOLR1- cells in normal samples (5.14% vs.72.6 %, 0.70% vs. 58.4%, and 0.83% vs. 55.1%, in CD45- cells respectively). It indicates that in non-malignant samples, both EPCAM and FOLR1 were markers of epithelial cells; while in tumor samples, tumor and normal epithelial cells had different epithelial markers (EPCAM for tumor epithelial cells and FOLR1 for normal epithelial cells), which may due to the heterogeneity of tumor cells. In summary, our FASC results proved the reliability of the tumor and normal lung markers in our study. As shown in Figure 10, we observed that the expression levels of ITGB1 (P < 0.01), LAMB1 (P < 0.01), CD70 (P < 0.01), and CD27 (P < 0.01) were significantly increased in LUAD tumor cells and the expression levels of CXCR5 (P < 0.01), CXCL13 (P < 0.01), ITGB4 (P < 0.01), and CCL5 (P < 0.01) were also increased prominently in tumor T cells. These findings were consistent with the scRNA-seq results, indicating that scRNA-seq data analysis can effectively explore the transcriptome of individual cells and the differentially expressed ligand-receptor pairs identified in scRNA-seq analysis exhibited significant changes in tumor samples.

## Discussion

Tumors are mixtures of different compartments and heterogeneity is well known as one of the most prominent characteristics of tumors 27. Previous studies have shown that a heterogeneous TME is likely to be closely associated with therapeutic outcome 3, 17, 28. Additionally, RNA-seq represents an average of gene expression in the sample, which may ignore significant and biologically differences between cells 29. Therefore, in RNA-seq technologies, the contribution of the TME is difficult to separate and the function of the TME can be confounded by the existence of non-neoplastic cells. However, compared with RNA-seq technologies, scRNA-seq allows investigation of the transcriptome of individual cells and enables exploration of the heterogeneous TME 30, 31.

In the present study, 36,095 cells from 10 samples of LUAD including six malignant samples and four normal samples were calculated in cluster analysis by PCA and UMAP clustering methods at first, and then the LUAD tumor cell and T cell clusters were identified by gene markers. To implement a more accurate classification, EPCAM, MDK, and SOX4; FOLR1, SFTPD, and AGR3; and CD3D, TRAC, and TRBC2 were used as specific markers for tumor cells, epithelial cells, and T cells, respectively, which have been verified and reported in many studies 32-37. We also found tumor samples contained 18.2% tumor cells and 53.4% T cells, while normal samples contained 10.4% epithelial cells and 44.1% T cells, indicating that T cells are the dominant cell type in tumor and normal samples. Besides, the gene expression of T cells has a significant effect on gene expression, consistent with previous studies 17, 28.

Many studies have reported that the number of tumor-infiltrating T cells that express checkpoint molecules such as PD-1, CTLA-4, and Lag-3 is a reliable prognostic marker in lung cancer and immune checkpoint therapy has served as an effective treatment strategy for LUAD 38, 39. The efficacy of immune checkpoint therapy may depend on the recruitment of tumor-infiltrating T cells, which is regulated by cell-cell interactions 40. For example, ligand-receptor chemokine pairs can activate intracellular signaling pathways and interfere with the recruitment of tumor-infiltrating T cells 41. Therefore, the present study may improve our understanding of cell communication and promote the identification of potential therapeutic targets in immunotherapy.

HLA-B and its ligand LILRB2 were found to be downregulated in LUAD tumor cells. LILRB2 is a negative regulator of myeloid cell activation and the expression of LILRB2 has been linked to cytoskeleton remodeling, metabolism, and endosomal sorting pathways, as well as changed differentiation gene networks associated with inflammatory myeloid cells 42, 43. Recent studies have revealed that LILRB2 blockade polarized tumor-infiltrating myeloid cells from non-small cell lung carcinoma (NSCLC) tumor tissues toward an inflammatory phenotype, and can potentially act as a myeloid immune checkpoint by reprogramming tumor-associated myeloid cells and provoking antitumor immunity 44. In the present study, the HLA-B-LILRB2 pair was the most downregulated. Meanwhile, 41 key upregulated and 55 key downregulated pairs were detected for T cell communication. The SELPLG-ITGB2 pair was found to be significantly increased. In a previous study, ITGB2 promoted the migration and invasion of breast cancer and activated integrin-related FAK signaling; however, there is a lack of research clarifying the function and mechanism of SELPLG-ITGB2 45. Overall, to explore the crosstalk between cancer cells and T cells, 119 ligand-receptor pairs, some of which could significantly affect survival outcomes, were confirmed in our research.

Finally, we built a prognostic model based on machine learning of these ligand-receptor genes and a GEO dataset including 1,063 patients was used to validate the model. In our prognostic model, overall survival was significantly different between high-risk and low-risk groups, and the genes ITGB4, CXCR5, and MET played an important role in prognosis. In NSCLC, MET pathway activation is thought to occur through a diverse set of mechanisms that influence properties affecting cancer cell survival, growth, and invasiveness. Preclinical and clinical evidence suggests a role for MET activation as both a primary oncogenic driver in subsets of lung cancer and as a secondary driver of acquired resistance to targeted therapy in other genomic subsets. Aberrant MET signaling can occur through many mechanisms, including MET or HGF protein overexpression, MET gene amplification, and MET gene mutation in downstream signaling or regulatory components 46, 47. More recent investigations focusing on MET exon 14 alterations and MET amplification have been notable for meaningful clinical responses to MET inhibitor therapy in a substantial proportion of patients 48, 49.

## Conclusion

Using scRNA-seq data, we provide and validate a landscape of intracellular communication and crosstalk in cancer cells and T cells of LUAD, respectively. We believe these observations will improve our understanding of the contribution of the TME to cancer progression and potential targets for immunotherapy in the future.

## Materials and Methods

### Ethics statement

This study was approved by the Ethics Committee of Zhongshan Hospital, Fudan University, China (B2018-137R). Informed consent was obtained when the patients were hospitalized.

### Datasets

Bulk tumor tissue RNAseq and scRNA-seq data of LUAD were downloaded from TCGA (https://tcgadata.nci.nih.gov/) and ArrayExpress (https://www.ebi.ac.uk/arrayexpress/) with accession number E-MTAB-6149 and E-MTAB-6653, respectively. Gene Expression Omnibus (GEO; https://www.ncbi.nlm.nih.gov/geo/) data (GSE30219, GSE31210, GES3141, GSE37745, GSE50081, and GSE68465) were downloaded as validation cohorts for the machine-learning model. Ligand-receptor pair information was obtained from the FANTOM5 project 50. Ten normal and ten LUAD samples were selected for quantitative real-time polymerase chain reaction (qRT-PCR) analyses. Part of the flow cytometry figures are shown in Supplementary Figure 8.

### Statistical analysis

#### The scRNA-seq data analysis

The scRNA-seq data analyses performed in R version 3.5.1 were as follows: (1) scRNA-seq data was converted as a Seurat object using the Seurat R package 51; (2) after quality control of data, the “FindVariableFeatures” function was used to find the top 1,500 highly variable genes; (3) based on these 1,500 genes, principal component analysis (PCA) and uniform manifold approximation and projection (UMAP) were performed to analyze the 10 scRNA-seq data; and (4) SingleR package 52, CellMarker dataset 53, and previous studies were used to recognize the different cell types obtained with scRNA-seq. And cells were removed if they met one of the following criteria: 1) the number of expressed genes lower than 101 or larger than 6000; 2) 10% or more of UMIs were mapped to mitochondrial or ribosomal genes.

#### Cell-cell communication analysis

After identifying the cell types in scRNA-seq, R package MAST 54 was applied to compare their expression between neoplastic and non-neoplastic cells. The statistical threshold for significance was at P < 0.05. To analyze intracellular communication, we first selected ligand-receptor genes that were both upregulated and downregulated in LUAD tumor cells and T cells. To further investigate the correlations in ligand-receptor pair genes, Spearman's correlation coefficients were calculated to verify the co-expression of ligand and its corresponding receptor genes. Co-expression of genes was considered with a threshold of coefficient > 0.4 and adjust P-values < 0.05 in Spearman's analyses. And “p.adjust” function in R was applied to calculate the adjust P-values.

We divided the data into two groups to investigate the crosstalk between T cells and LUAD tumor cells: (1) ligands and receptors that were separately highly expressed in T cells and LUAD tumor cells were selected; and (2) ligands highly expressed in LUAD tumor cells and receptors highly expressed in T cells were selected. Gene functional enrichment analysis was then applied to discover the function of related genes.

### Machine learning model

Extreme Gradient Boosting (XGBoost) is a decision-tree-based ensemble Machine Learning algorithm, which was conducted based on the Gradient Boosting framework. Compared with other machine-learning models, XGBoost improves upon the base Gradient Boosting framework through systems optimization and algorithmic enhancements. Furthermore, to solve prediction problems effectively, XGBoost provides a parallel tree boosting to achieve state-of-the-art results 55. To build a predictive model, the TCGA LUAD dataset was split into low risk (stage IA, 127 cases) and high risk (stage IB-IV, 333 cases) groups, then these patients were served as prediction labels to train our prediction model. PCA was performed to determine whether there were interior differences in the expression of the ligand-receptor pairs in these two types. We used the “sample” function in R software to randomly divide the TCGA dataset into training and test sets with a 3:1 ratio; the python package 'sklearn' was performed to construct a machine learning model and XGBoost was applied to train the model to explore the important genes in cell to cell communication and predict clinical outcomes. Patients (without AJCC stages information) from the GEO dataset were used as a validation cohort to test our model.

### Survival statistical analysis

Kaplan-Meier and log-rank tests were used to construct and compare survival curves. To confirm whether the selected genes were associated with poor survival, we split the patients into a high expression group (> median expression level across all samples) and a low expression group (≤ median expression level across all samples). A significant difference in survival analysis was defined as P < 0.05. Survival statistical analyses were performed in R.

### Validation

Single cells were suspended in phosphate-buffered saline with 3% fetal bovine serum and incubated with 20 μg/mL human IgG (Sigma-Aldrich, St. Louis, MO, USA) for 15 min to block nonspecific antibody binding. Subsequently, cells were incubated with allophycocyanin-conjugated mouse anti-human EPCAM (5 µL/106 cells; cat. no.: 566658, BD Biosciences, San Jose, CA, USA), BV421-conjugated mouse anti-human CD45 (5 µL/106 cells; cat. no.: 304022, BioLegend, San Diego, CA, USA), FITC-conjugated mouse anti-human CD3D (5 µL/106 cells; cat. no.: MHCD0301, ThermoFisher Scientific, Waltham, MA, USA), PE-conjugated mouse anti-human FOLR1 (10 µL/106 cells; cat. no.: FAB5646P, R&D Systems, Minneapolis, MN, USA) for 30 min on ice. Then, the Fortessa analyzer (BD Biosciences) and FACSAria II (BD Biosciences) were used to quantitate and isolate stained cells, respectively. FlowJo software (TreeStar, Woodburn, OR, USA) was used to generate the flow described above. Fortessa analyzer (BD Biosciences) and FACSAria II (BD Biosciences) were used to quantitate and isolate stained cells, respectively. FlowJo software (TreeStar, Woodburn, OR, USA) was used to generate the flow described above. In qRT-PCR analyses, sorted cells were subjected to RNA extraction and reverse transcription using a kit (Takara, Kusatsu, Japan) before the experiment. In RT-qPCR analyses, sorted cells were subjected to RNA extraction by TRIzol (Beyotime, China). PrimeScript RT reagent Kit with gDNA Eraser (Real Time Perfect) (Takara, Kusatsu, Japan) was used to synthesized the first-strand cDNA. Then with the proper PCR parameters (1 cycle of 30 s at 95°C, 40 cycles of 5 s at 95°C and 34s at 60°C), SYBR Premix Ex TaqTM II (Tli RNaseH Plus) (TaKaRa) was applied in our study. β-actin was used as the reference. Primers used in this study are listed in Supplementary Table 1.

## Supplementary Material

Supplementary figures and Supplementary table 1.

Supplementary table 2.
