# Supplementary material for: Ligand-receptor interaction atlas within and between tumor cells and T cells in lung adenocarcinoma
Source: Int J Biol Sci. 2020 May 18;16(12):2205–19. doi: 10.7150/ijbs.42080 (PMC7294944; doi:10.7150/ijbs.42080)
Supplement: Supplementary file 1 — Supplementary figures and Supplementary table 1. [file ijbsv16p2205s1.pdf]

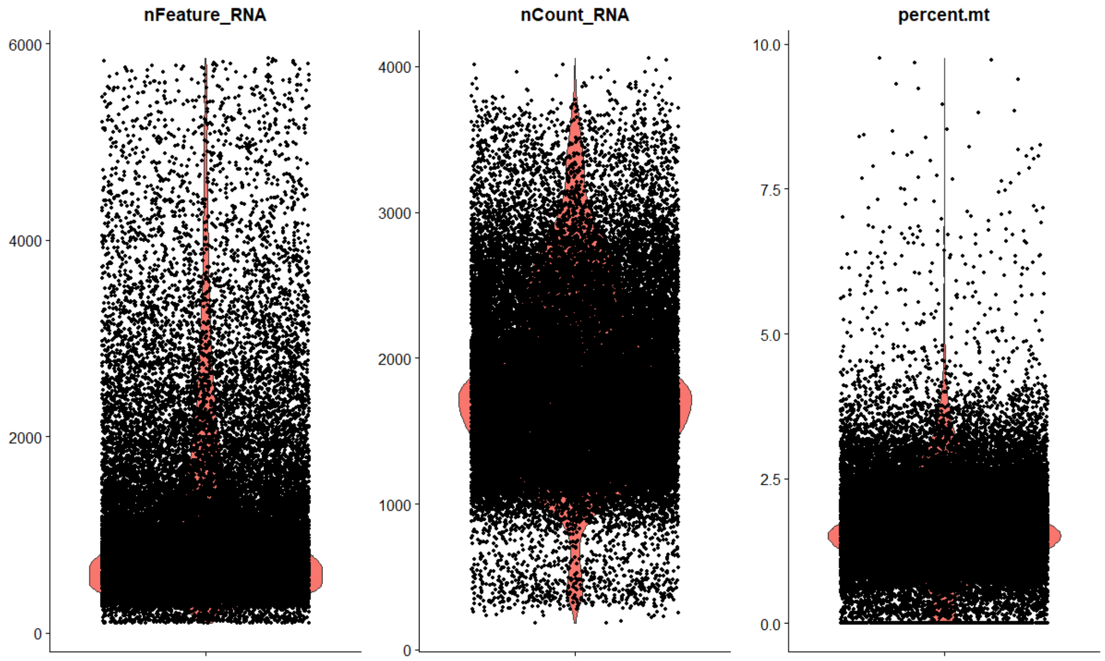

Supplementary Figure 1. Quality control of scRNA-seq for all cell sub-populations.

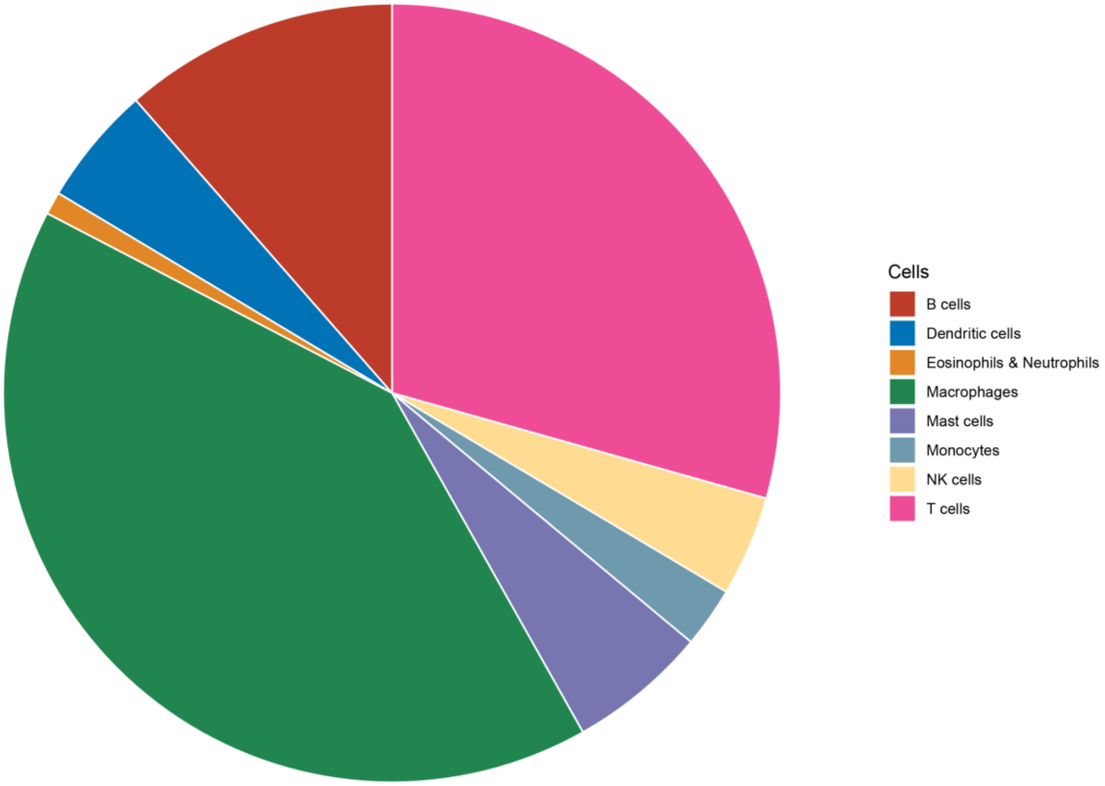

Supplementary Figure 2. Result of CIBERSORT analysis.

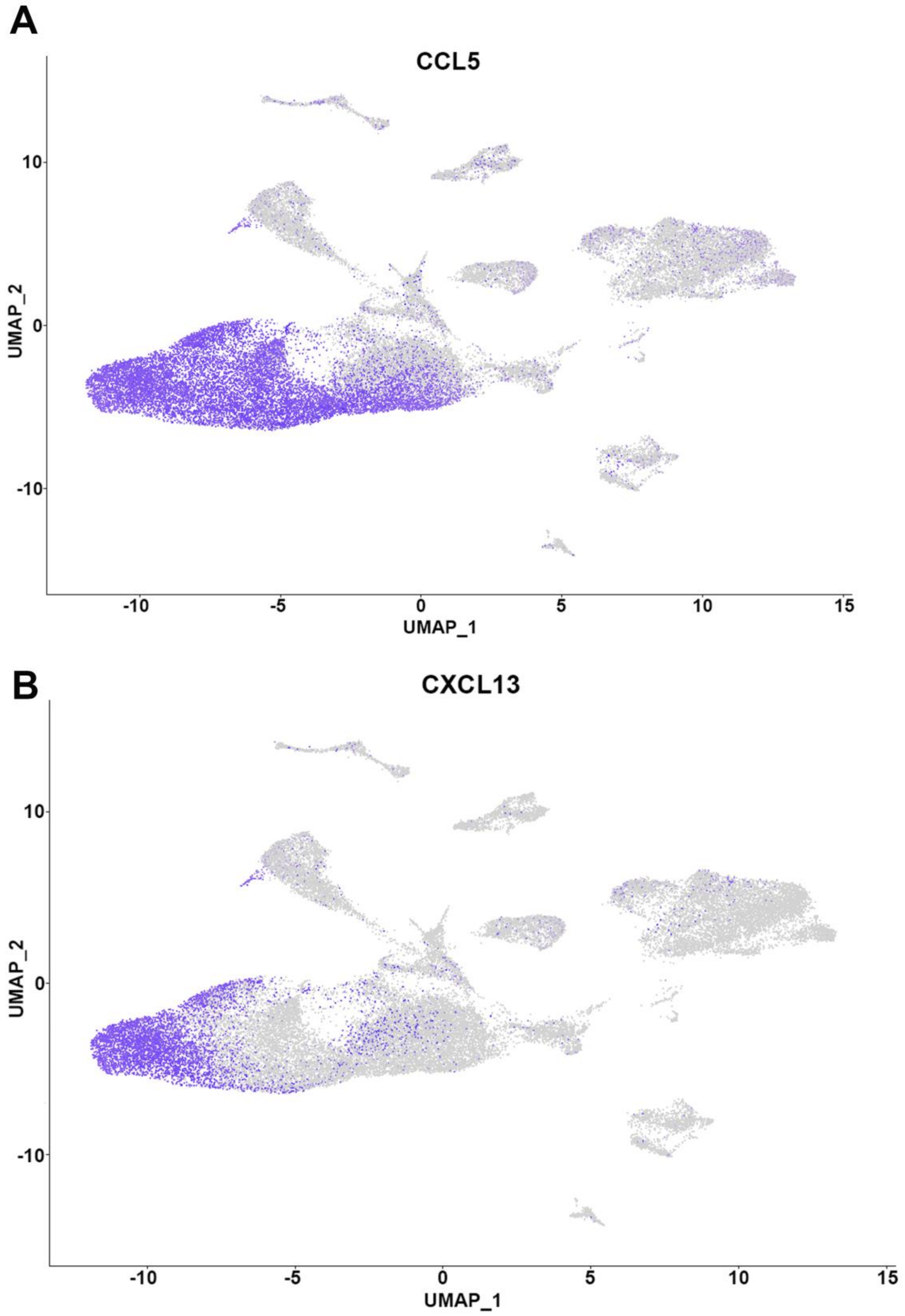

Supplementary Figure 3. Feature plot of CCL5 and CXCL13.

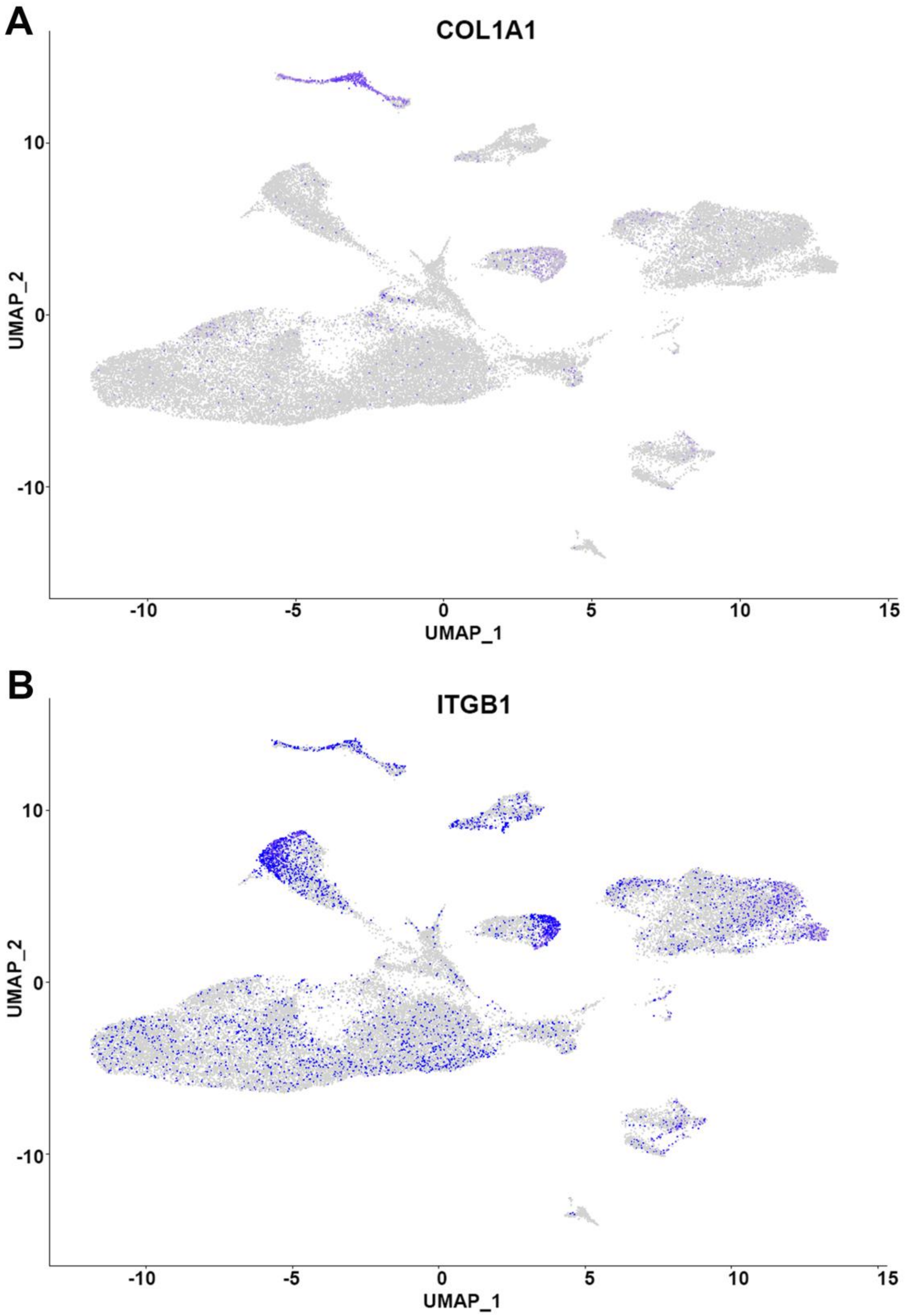

Supplementary Figure 4. Feature plot of COL1A1 and ITGB1.

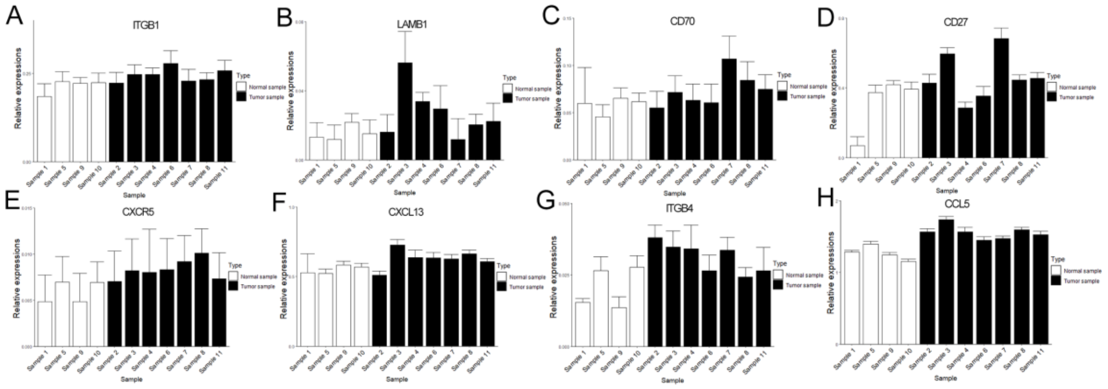

Supplementary Figure 5. Compared the key genes expression in epithelial cells (LUAD tumor cells and Alveolar cells) and T cells (Tumor T cells and Non-tumor T cells) among different samples. (A-D) Compared the expression level of ITGB1, LAMB1, CD70, and CD27 in LUAD tumor cells; (E-H) Compared the expression level of CXCR5, CXCL13 ($P < 0.01$), ITGB4, and CCL5 in T cells.

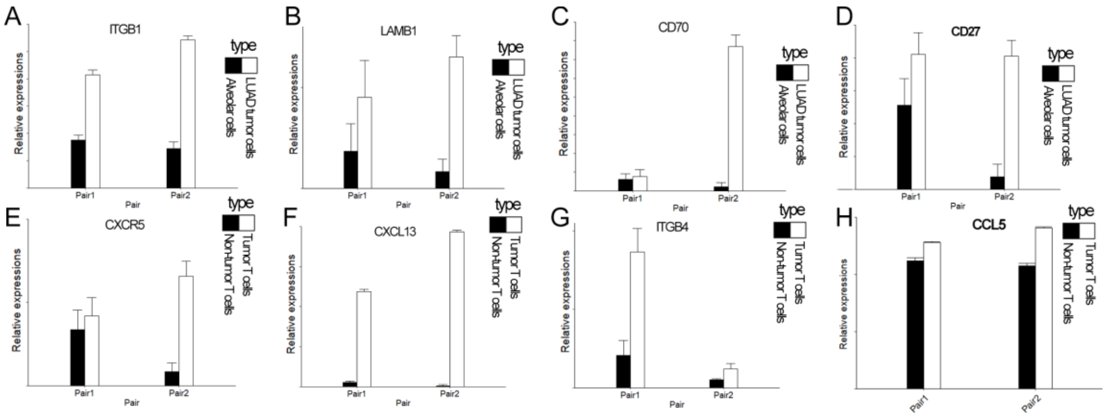

Supplementary Figure 6. Compared the expression of ITGB1, LAMB1, CD70, CD27, CXCR5, CXCL13, ITGB4, and CCL5 in the pairs of tumor and corresponding normal samples. (A-D) Compared the expression level of ITGB1, LAMB1, CD70, and CD27 in LUAD tumor cells; (E-H) Compared the expression level of CXCR5, CXCL13 ($P < 0.01$), ITGB4, and CCL5 in T cells.

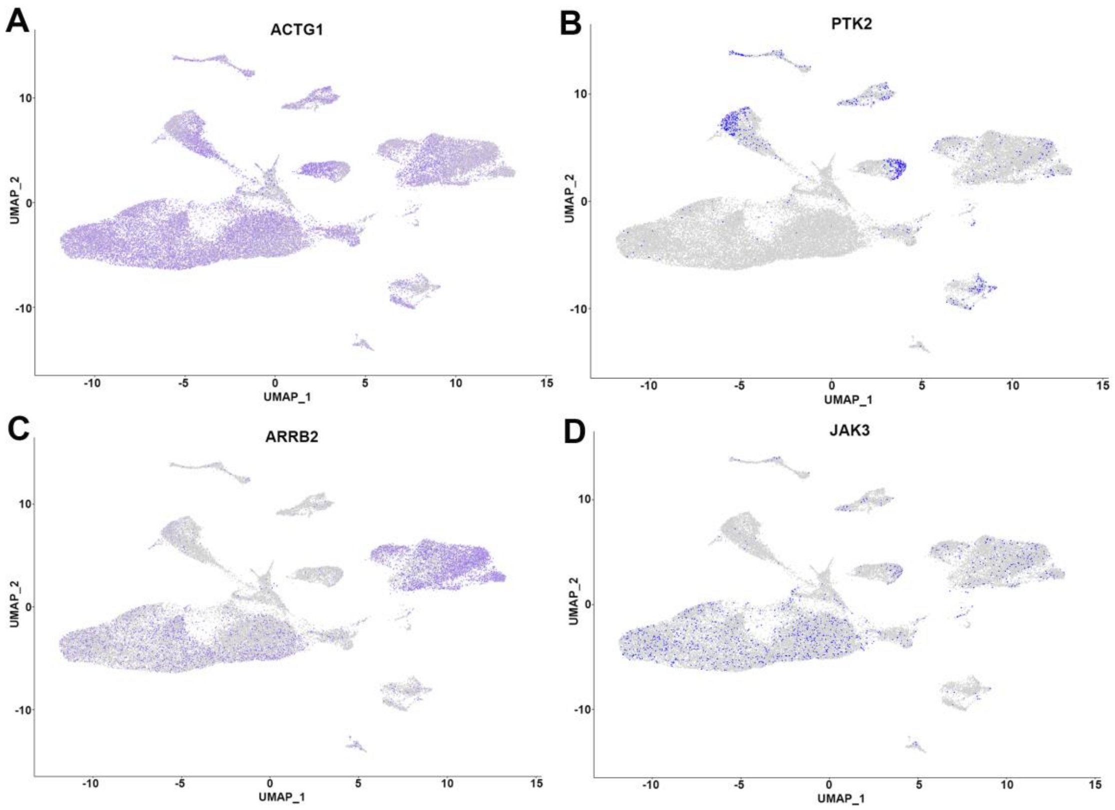

Supplementary Figure 7. Feature plot of ACTG1, PTK2, ARRB2, and JAK3.

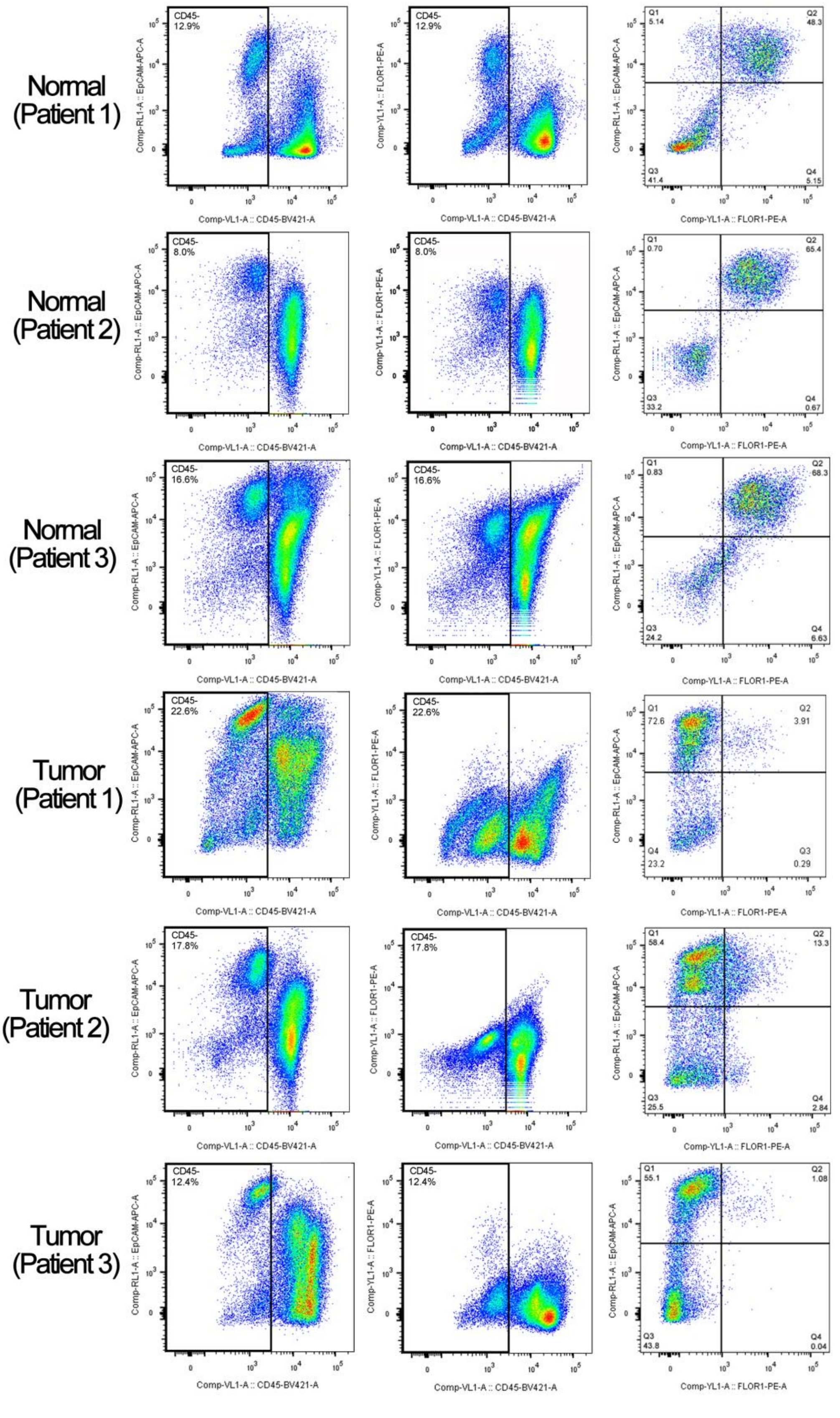

Supplementary Figure 8. Three pairs of normal/ tumor LUAD samples in double-positive cells in FACS analysis (Left: CD45-EPCAM+ cells, Middle: CD45-FOLR1+ cells, Right: EPCAM+FOLR1+ cells).

Table I Characteristics of the 5 patients included in this study for scRNA-seq analysis

	Age	Sex	Stage	Location	TNM	Smoking status	COPD/GOLD
1	86	Male	IB	RUL	pT2bN0M0	Former	Yes (2)
2	68	Male	IIIB	RUL	pT4N2M0	Former	No
3	64	Female	IIB	LUL	pT2aN1M0	Former	Yes (2)
4	60	Male	IA3	LUL	pT1cN0M0	Former	Yes (2)
5	65	Male	IIIA	LUL	pT4N1M0	Former	Yes (2)

Tables II Characteristics of the 11 lung tissue samples included in this study

Sample	Patient	Sample Type
1	1	Normal
2	2	LUAD
3	2	LUAD
4	2	LUAD
5	2	Normal
6	3	LUAD
7	3	LUAD
8	3	LUAD
9	3	Normal
10	4	Normal
11	5	LUAD

LUAD： Lung adenocarcinoma
RUL: Right upper lobe
RLL: Right lower lobe
RML: Light middle lobe
LLL: Left lower lobe
LUL: Left upper lobe

Primers used in this study

ITGB1-forward: 5'-TTGACCCTAATACCAACCTTCCT-3';

ITGB1-reverse: 5′-CCCATAGTACAGCCCTTGATGTT-3′;

LAMB1-forward: 5'- AGGAACCCGAGTTCAGCTAC-3';

LAMB1-reverse: 5′- CACGTCGAGGTCACCGAAAG-3′;

CXCR5-forward: 5′-CACGTTGCACCTTCTCCCAA-3′;

CXCR5-reverse: 5′-GGAATCCCGCCACATGGTAG-3′;

CXCL13-forward: 5′- GCTTGAGGTGTAGATGTGTCC-3′;

CXCL13-reverse: 5′- CCCACGGGGCAAGATTTGAA-3′;

CD70-forward: 5′-GCTTTGGTCCCATTGGTCG-3′;

CD70-reverse: 5′-CGTCCCACCCAAGTGACTC-3′;

CD27-forward: 5′- CAGAGAGGCACTACTGGGCT-3′;

CD27-reverse: 5′- CGGTATGCAAGGATCACACTG-3′;

CCL5-forward: 5′- CCAGCAGTCGTCTTTGTCAC-3′;

CCL5-reverse: 5′- CTCTGGGTTGGCACACACTT-3′;

ITGB4-forward: 5′-GCAGCTTCCAAATCACAGAGG-3′;

ITGB4-reverse: 5′-CCAGATCATCGGACATGGAGTT-3′.
